# Structural Design and Simulation of Multi-Detector Same-Platform Laser Gyro Reflector Substrate Defect Detection Prototype

**DOI:** 10.3390/mi15121498

**Published:** 2024-12-15

**Authors:** Jun Wang, Zhenyang Li, Maoxin Song, Zhilong Xu, Huan Luo, Mingchun Ling, Hengwei Qin, Wuhao Liu, Zhenhai Liu, Jin Hong

**Affiliations:** 1 University of Science and Technology of China, Hefei 230026, China; sa21168104@mail.ustc.edu.cn (J.W.); renliu0619@mail.ustc.edu.cn (H.Q.); liuwuhao@ustc.edu (W.L.); hongjin@aiofm.ac.cn (J.H.); 2 Hefei Institutes of Physical Science, Chinese Academy of Sciences, Hefei 230031, China; zlxu@aiofm.ac.cn (Z.X.); luohuan@aiofm.ac.cn (H.L.); lingmingchun@126.com (M.L.); lzhenhai@aiofm.ac.cn (Z.L.); 3 Key Laboratory of Optical Calibration and Characterization, Chinese Academy of Sciences, Hefei 230031, China

**Keywords:** laser gyro, fusion of light and dark fields, patterned and unpatterned defect detection, multi-detector same platform, structural design

## Abstract

Defect detection and classification in super-high reflector mirrors and their substrates are crucial for manufacturing laser gyroscope systems. This paper presents a prototype designed to meet the requirements for the reflection and transmission of laser gyroscope mirror substrates. The prototype featured two measurement channels (bright field and dark field) and could detect defects on patterned and unpatterned surfaces. Key components were simulated using Ansys software, (Ansys Workbench 2022 R1)which showed a maximum static deformation of 4.65 μm, a resonant frequency of at least around 230 Hz, and a maximum stress of 9.86 MPa under transportation conditions (GJB150.16A-2009). These results confirm the prototype’s stability for optical performance testing and structural design. The experimental testing on laser gyroscope reflector substrates and USAF 1951 plates demonstrated that the prototype effectively detected defects on reflection and transmission surfaces, with a detection resolution that exceeded 170 nm, which met the design requirements.

## 1. Introduction

A laser gyro serves as a high-precision inertial sensor primarily employed for detecting an object’s angular motion. Its core component consists of ring-shaped lasers arranged in quadrilateral or triangular configurations [1]. When integrated with accelerometers to form inertial navigation systems, a laser gyro facilitates the precise spatial position determination of moving carriers without reliance on a GPS [2], rendering them indispensable components in the aviation, aerospace, and maritime domains. The laser gyro’s reflector substrate, characterized by ultra-high reflectivity, stands as a precision optical device within the gyroscope system. During the manufacturing process, surface defects, including speckles, scratches, open bubbles, voids, and edge fractures [3], that significantly affect the surface quality often appear. Small surface defects, influenced by size effects [4] and surface reflection [5], induce substantial light scattering and loss, leading to lock-in effects and increased random drift in the laser gyro [6]. Larger surface defects, such as long scratches, absorb considerable light energy, generating thermal stress and affecting the laser phase [3]. With the escalating demands for inertial guidance system accuracy, current reflectivity requirements for laser gyro reflectors have reached 99.99% or higher, magnifying the detrimental impact of minor defects on laser gyro performance.

Initially, the process of identifying defects in laser gyroscope reflector substrates relied heavily on manual visual inspection. However, given its limited productivity and inability to maintain consistent accuracy [7], this method is steadily being phased out and replaced by automated defect inspection (ADI) methodologies [8,9]. There exists a diverse array of technologies for detecting defects, broadly classified into non-contact and contact-based methods. Among the commonly used contact-based techniques, profilometers and atomic force microscopes (AFMs) are frequently employed, albeit they tend to cause secondary damage to the component’s surface [10]. Non-contact inspection methods include optical and non-optical techniques. For non-optical inspection, electron microscopy (SME), ultrasonic testing (UT), and infrared thermography are prevalent. However, these methods have limitations, such as detection accuracy or being limited to specific surface conditions, which makes them unfit for large-scale industrial production [4]. Conversely, automated optical inspection (AOI) technology has emerged as the predominant trend in defect detection due to its advantages of speed, non-contact nature, non-destructiveness, and ease of integration into online systems [11,12].

Currently, common AOI techniques include interferometry, diffraction, and scattering methods. Interferometric detection methods encompass white-light interferometers and laser interferometric profilometers; however, these methods suffer from issues such as complex adjustment and limited lateral resolution [13]. The diffraction-based detection method relies on the diffraction pattern formed on the plane after transmission to establish a relationship with the defects’ shapes and sizes; however, this approach is only suitable for regular defects [14,15]. Hence, these two methods have not been widely applied in defect detection. Since defects produce abrupt changes in the refractive index, exhibiting scattering characteristics, scattering methods utilize the defects’ scattering effects on incident light as widely used defect detection approaches [16]. Common methods of scattering defect detection are divided into bright-field and dark-field systems, which differ significantly in their illumination methods, imaging principles, and corresponding technical difficulties. Due to its high contrast, dark-field systems can detect defects far smaller than the system resolution, improving the system sensitivity [17]. Additionally, dark-field systems offer sufficient detectability for micro-roughness and imperfections within crystals [18]. The bright-field system sensitivity is comparable with its system resolution, and even for macroscopic defect detection, the bright-field system is clearer and more intuitive [17]. Overall, bright-field systems impose stricter requirements on the physical characteristics of the illumination beams, imaging systems, signal-to-noise ratios, etc. [19]. Typically, dark-field systems are used for defect detection without patterns, while bright-field systems or their combination are employed for patterned defect detection [20,21].

Based on the above introduction, to achieve the simultaneous surface defect detection in transmission and reflection, this paper presents the following research: First, it introduces the optical detection principles of the defect detection prototype, followed by a description of the prototype’s structural design and implementation, as well as the static, modal, and random vibration simulations. The prototype’s assembly and tuning processes are then described, culminating in the actual product demonstration. Finally, experimental verification was conducted regarding the instrument’s operating mode and defect detection capability. The experimental results indicate that the prototype’s detection resolution exceeded 170 nm. Moreover, the design fulfilled the simultaneous detection requirements of high-transmission and high-reflection surfaces, as well as integrated the simultaneous surface and edge detection functionalities. This design can provide assurance for high-precision laser gyroscope manufacturing.

## 2. Materials and Methods

### 2.1. Optical Principle

**Figure 1:**
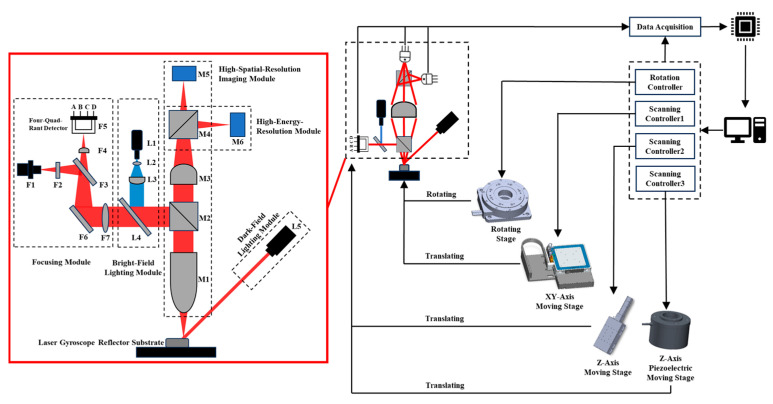
Schematic diagram of the optical principle. Focusing module: laser diode F1, grating F2, splitter F3, cylindrical astigmatism lens F4, four-quadrant detector F5, reflector F6, collimating lens F7; lighting module: LED light source L1, uniform light film L2, condenser lens L3, dichroic filter L4, laser (635 nm) L5; microscopic detection module: objective lens (20×) M1, first beam splitter cube M2, tube mirror M3, second beam splitter cube M4, CMOS detector M5, photomultiplier tube M6.

The experimental setup was based on a planar scanning methodology, wherein the detection surface traverses the designated test area by displacing the specimen under examination. This methodology facilitates the defect detection on the surface and periphery of the reflector substrate employed in the laser gyro system, as depicted schematically in Figure 1. Motion apparatus control by the host computer orchestrates the optical instrument’s scanning and focusing operations. Subsequently, the test sample translation along a predetermined trajectory is orchestrated by the loading platform manipulation. The conversion of each detection surface signal into an electrical counterpart is executed by the detector. Afterward, these signals are transmitted to the main computer through a data collector for subsequent image-processing stages.

The optical inspection segment was functionally subdivided into the focusing measurement, illumination, and microscopic imaging modules. It encompasses two distinct measurement channels: bright field and dark field, along with two detection modes: patterned and non-patterned. Additionally, it operates under two distinct working conditions: surface inspection and edge detection.

In the focusing module, the focusing laser diode emits a laser beam (655 nm) that passes through a grating and beam splitter before being directed onto the sample surface. Diffraction gratings modulate the laser beam’s amplitude and phase, enabling them to focus with smaller spot sizes and improving the focusing accuracy. In addition, its diffraction grating can reduce the light beam’s scattering and reflection on the optical disc’s surface, thereby improving the signal’s clarity and accuracy. After the reflection, the beam returns through a cylindrical astigmatic lens to a quadrant photodetector. By rapidly scanning the *Z*-axis stage or piezoelectric stage up and down, the energy values of the four quadrants (*A*, *B*, *C*, and *D*) are recorded. The focusing signal intensity is calculated using Equation (1). Utilizing the different focal positions for the sagittal and tangential images in the astigmatic lens, the optical–mechanical system is at the focal plane when the signal curve matches the expected curve and there is a unique zero signal value.

(1) $$\mathrm{Focusing}\;\mathrm{signal}=\left(E_{A}+E_{C}\right)-\left(E_{B}+E_{D}\right)$$

where *Ei* ∈ {*A*, *B*, *C*, *D*} represents the signal strength received by the four quadrants of the four-quadrant detector.

In the microscopic detection module, the system comprises a microscope objective, a tube lens, two beam-splitting cubes, and dual detectors (CMOS + PMT). These components are aligned along the laser illumination’s normal direction and coaxially with the LED illumination. The microscopic detection module’s two detectors were placed in conjugate positions via the second beam-splitting cube, enabling the simultaneous acquisition of defect images and energy signals.

For dark-field illumination, a laser (635 nm) is obliquely incident on the sample surface. The microscopic detection system (e.g., a high NA objective) collects the scattered light caused by defects within a specific aperture in the normal direction and focuses it onto the CMOS detector and photomultiplier tube (PMT). The presence of defects is indicated by a rapid increase in signal due to illumination light scattering.

For bright-field illumination, a blue LED (460–470 nm) with a certain divergence angle is used. Coaxial illumination is achieved through a diffuser, condenser lens, dichroic filter, and beam-splitting cube to illuminate the inspected sample. The dichroic filter reflects light in the 385–520 nm range and transmits light in the 565–1200 nm range, allowing the focusing module laser to pass through the optical path without interference from the illumination source, ensuring accurate energy collection by the focusing module’s quadrant photodetector.

### 2.2. Structural Design

**Figure 2:**
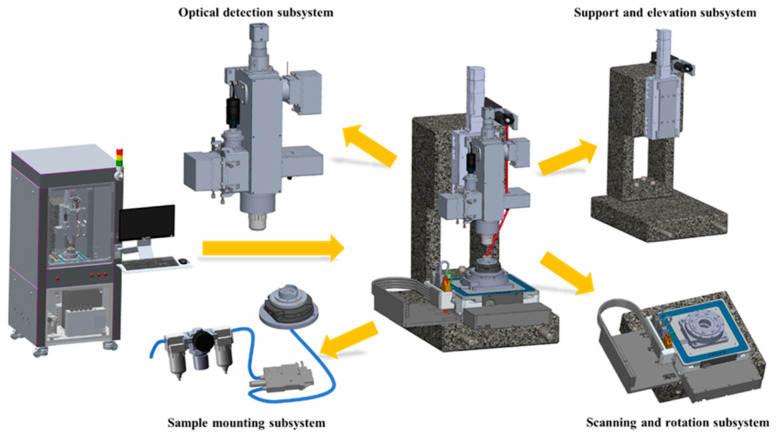
The structure of the laser gyro reflector substrate defect inspection prototype.

Based on the aforementioned optical principles, the structural design of the prototype for defect detection on the laser gyro’s reflector substrate (Figure 2) consists of four subsystems: optical detection, sample mounting, support and elevation, and rotation and scanning.

#### 2.2.1. Optical Inspection Subsystem

Based on the principles illustrated in Figure 1, the subsystem comprises a focusing measurement, illumination, and microscopic detection modules. In accordance with optical principles, the optical inspection subsystem needed to simultaneously meet the following requirements to achieve a minimum detection resolution of 170 nm for the prototype. Additionally, we ensured each module operated optimally to leverage the advantages of the system’s multiple detectors and dual illumination.

1. The focal points of the focusing measurement module and the microscopic detection module needed to coincide to ensure the reception of a centrally positioned circular spot on the quadrature detector.
2. When the system was in the focal plane position, the lighting module illumination area needed to be not less than 0.71 mm × 0.85 mm.
3. In accordance with the microscopic detection module requirements, the CMOS detector and PMT needed to be positioned at conjugate locations to ensure the simultaneous detection of the strongest signal at the same location.
4. The focal points of the bright-field LED illumination, dark-field laser illumination, and objective lens needed to coincide to ensure consistency in the dual-source detection.

**Figure 3:**
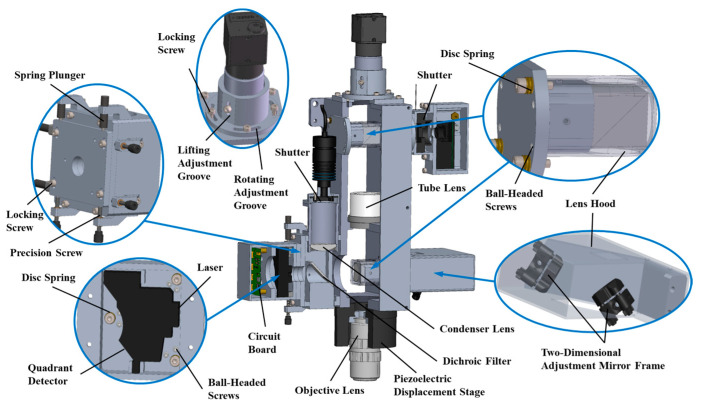
Structure diagram of optical detection subsystem.

The structural design scheme that satisfied the aforementioned indicators is depicted in Figure 3. In the focusing measurement module, a modified DVD read/write head (HOP-1200W-B) and a signal amplification circuit board for the quadrature detector were combined to adjust the received light for an optimal astigmatic condition. The positions of the laser diode and the quadrature detector affect the spot shape formed on the quadrature detector by the reflected light. This includes the angular misalignment and deviations in the vertical, horizontal, and lateral directions. Therefore, it was necessary to design a mechanism for adjusting the angular misalignment and two-degrees-of-freedom translation for the focusing measurement module. The angular misalignment adjustmentis achieved using a combination of disc springs and ball-headed screws, utilizing the principle of top pull. The two-degrees-of-freedom translation adjustment is realized with spring plungers and precision screw threads.

In the illumination module, a point light source from bright-field LEDs, after uniformization and focusing, is coaxially directed onto the specimen via dichroic filters and beam splitter cubes. Similarly to the focusing measurement module, the beam splitter cubes are adjusted using the top-pull method to ensure a uniform and sufficiently large illumination area on the specimen surface, while additional light shields were installed to minimize the influence of stray light. For dark-field illumination, the laser beams are directed onto the specimen surface from an oblique angle via deflecting mirrors. Each deflecting mirror required a two-degrees-of-freedom adjustment to ensure the adjustability of the laser beam angle and spot position.

In the microscopic detection module, the tube lens was mounted on the front of the objective lens to ensure a sufficient collection aperture angle, thus boosting the scattered signals’ intensity and enhancing the detection sensitivity. Both the CMOS detector and the PMT required circumferential and radial adjustment capabilities to ensure that the dual detectors are positioned at the tube lens’s focal point and are mutually conjugate. The adjustment method for the beam splitter cube is identical to that of the illumination module.

#### 2.2.2. Sample Mounting Subsystem

The angular adjustment platform was employed to level the test sample, while the sample mounting platform was utilized for circumferential sample limitation to ensure its proper positioning. Additionally, a step design was incorporated to accommodate various reflector substrate types for laser gyroscopes. Furthermore, a vacuum generator-based suction air channel serves as the key component to produce the necessary vacuum environment for securing the sample.

The nos. 1–6 flues of the entire vacuum adsorption platform are connected. The nos. 1–4 flues are connected to the sample mounting platform vacuum slots. Through the stepped design of the sample mounting platform, samples of different sizes can be securely fixed without the need to replace the mounting platform. Air duct 6 is connected to the vacuum channel via an air pipe quick-insert rotary head accurately positioned on the platform. This ensures unimpeded airflow in the vacuum channel and sample stability. The platform and rotating platform alignments needed to be coaxial, with dowel pins used for coarse positioning to prevent issues such as defocusing and missed detection [22].

**Figure 4:**
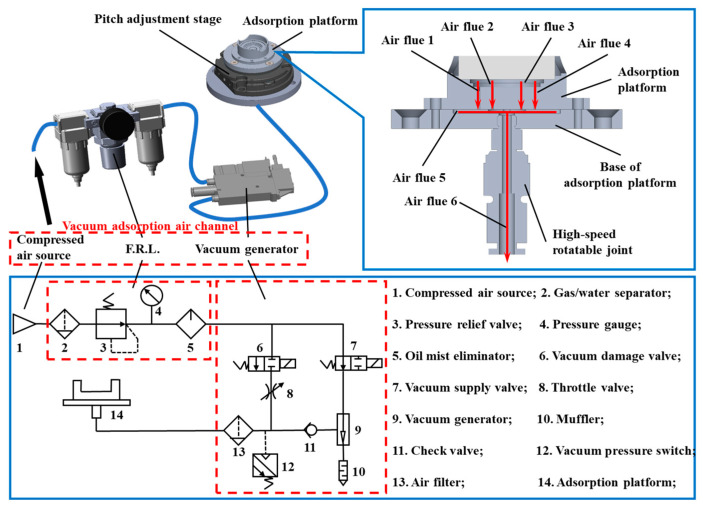
Schematic diagram of the sample mounting subsystem air path.

To safeguard the sample from harm caused by excessive impact during both adsorption and release, it was necessary to design a vacuum channel (Figure 4). The F.R.L. was utilized to remove particles and oil contaminants from the air. The vacuum generator (SMC, Japan, maximum vacuum degree −90 kPa) was utilized to create a vacuum environment. The airflow direction in the channel is managed using vacuum activation and deactivation valves (both being electromagnetic). Upon opening the vacuum activation valve and closing the vacuum deactivation valve, the vacuum producer becomes linked to the compressed air, slowly removing air from within the chuck to establish a vacuum, resulting in the chip being held by suction. Alternatively, if the vacuum release valve is open while the vacuum supply valve is shut, the chuck becomes linked to the compressed air through a throttling valve, which removes the vacuum inside and allows the chip to be released progressively. This seamless adsorption and release process minimizes the stress applied during handling.

#### 2.2.3. Support and Elevation Subsystem

**Figure 5:**
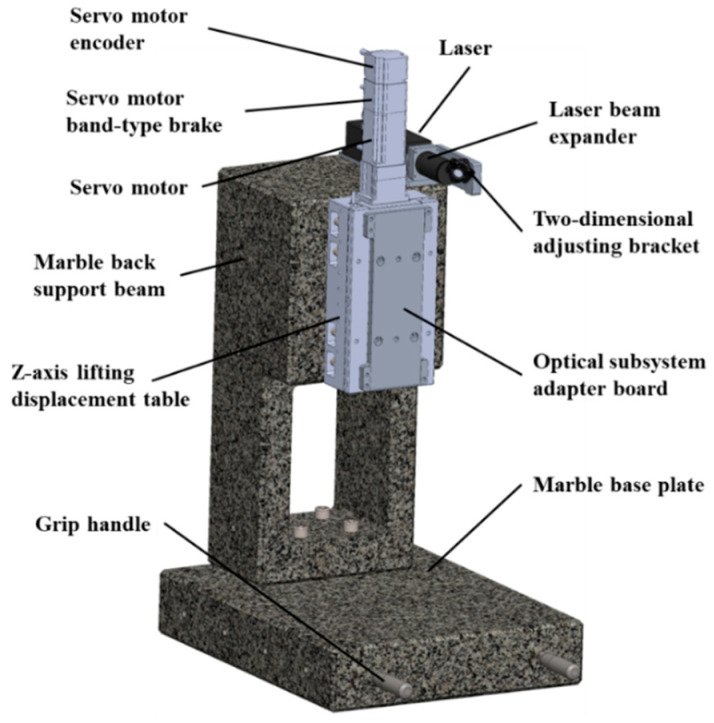
Support and elevation subsystem.

The scanning and focusing implementations rely on the support and elevation subsystem as the central element, while also facilitating spatial movement for the optical inspection subsystem. Given the optical inspection subsystem integration, the selected lifting platform necessitated a brake system. Moreover, the prototype’s detection performance is directly influenced by the movement precision and structural stiffness of the support and lifting subsystem. Thus, as illustrated in Figure 5, it was imperative to design a highly stable instrument base and select the lifting platform judiciously.

The support and elevation subsystem utilizes a marble fixed beam configuration to support the lifting platform, which is moved by the optical engine system. Simultaneously, the lifting platform is equipped with a brake electromagnetic coil, encoder feedback signals, and a drive motor. The brake electromagnetic coil is employed to brake the motor, preventing collision or overspeed situations between the motor carrying the optical inspection subsystem and the sample. The lifting platform (Akribis, Singapore) is equipped with grating rulers, providing feedback signals through encoders to the main control computer to determine the motor positioning accuracy. The drive motor (rated power of 0.1 kW, rated torque of 0.32 Nm, maximum torque of 0.96 Nm, rotor inertia of 0.5 × 10^–5^ kg·m^2^) is responsible for driving the lifting platform’s motion. Additionally, this structure required necessary mechanical simulations, the details of which are discussed in Section 2.3.

#### 2.2.4. Rotation and Scanning Subsystem

**Figure 6:**
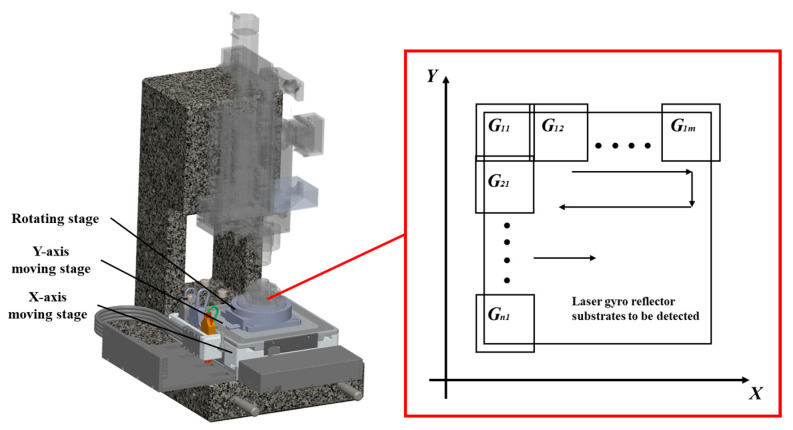
Scanning and rotation subsystem and working geometry schematic diagram.

The rotation and scanning subsystem serve as the central element for executing scanning motion, providing high-precision spatial movement for image scanning. To meet the requirements of wide-ranging image presentation and multiple angles, the operation of the scanning subsystem, along with its associated sample holder subsystem, needed to conform to the operation principle illustrated in Figure 6. For scanning imaging, a 10% overlap was set between each pair of consecutive images to prevent edge defect omissions. Under dark-field laser illumination, scratch-type defects exhibit sensitivity to light normal to their surface while being insensitive to light entering radially, which necessitated using a rotating platform for multi-angle imaging. The motion precision in the rotation and scanning subsystem directly determines the prototype’s detection capability. Hence, appropriate displacement stages (Akribis, Singapore) with a minimum resolution of 0.1 μm, a travel range of 110 mm, and a repeatable positioning accuracy of no less than ±0.5 μm, as well as rotational stages (Akribis Singapore) that could reach a maximum speed of 400 rpm, with a maximum radial and axial deviation of 5 μm, and a repeatable positioning accuracy of no less than ±4 arc sec, were selected to drive the samples.

The XY scanning displacement table moves linearly according to the path planning in Figure 6, while the rotating table moves nonlinearly and rotates 45° for the next azimuth detection after the scanning is completed. After the rotation is completed, the previous XY scanning table movement is repeated.

### 2.3. Simulation

#### 2.3.1. Static Mechanics Simulation

A static mechanics simulation was performed to confirm the mechanical properties when subjected to its own weight, with an emphasis on verifying the integrity of the instrument base and optical inspection subsystem. Simplified models were imported into Ansys, and the automatically generated connection methods were adjusted according to the actual conditions. A linear elastic material model was employed, with the material definitions outlined in Table 1.

**Figure 7:**
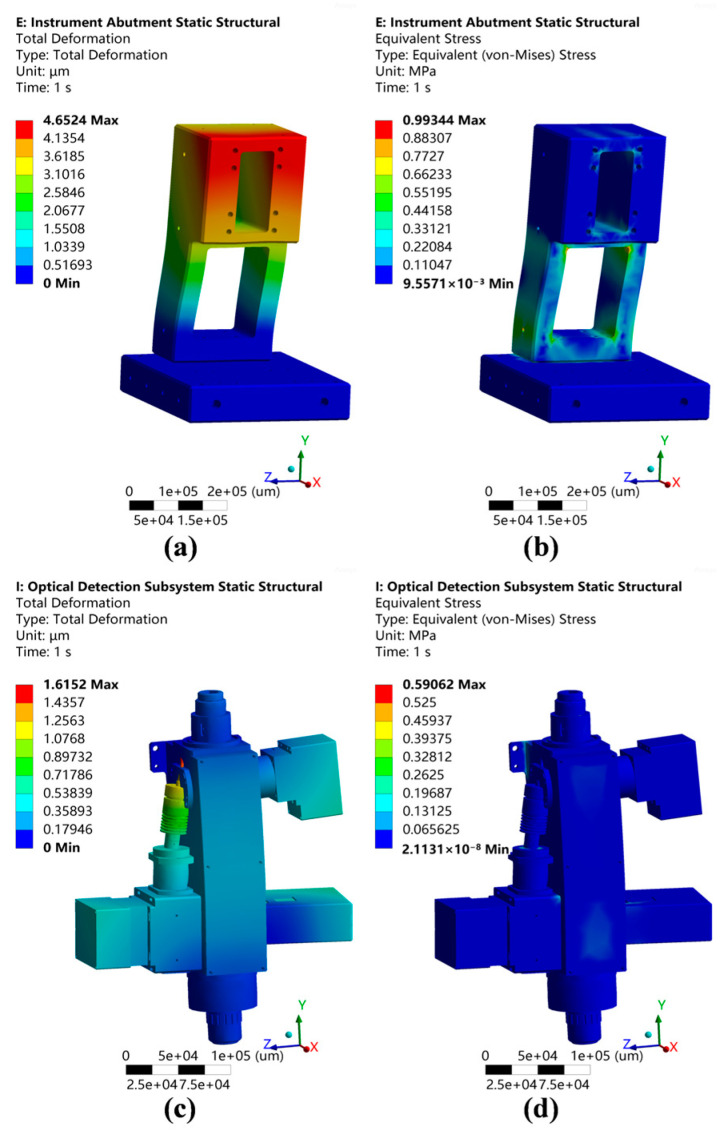
Static simulation results. (**a**) Instrument base deformation nephogram. (**b**) Equivalent instrument base stress nephogram. (**c**) Optical detection subsystem deformation nephogram. (**d**) Equivalent optical detection subsystem stress nephogram.

Certain components were simplified as mass points, and the mesh was automatically generated using Ansys, followed by localized adjustments. Gravity effects were incorporated, and fixed supports were added at the structural mounting locations. The simulation results are depicted in Figure 7.

From the simulation results, it is evident that the maximum instrument base deformation (4.65 μm) occurred at the lifting platform mounting location, while the peak distortion within the optical inspection subsystem (1.61 μm) was observed at the bright-field LED illumination location. The instrument base deformation level was less than 10 μm, which can be compensated for by the lifting platform for a focusing adjustment and the horizontal displacement platform for a lateral movement. Critical component deformations in the optical inspection subsystem may affect the instrument’s overall detection performance, as indicated by the simulation results presented in Table 2 showing that all the critical components exhibited vertical deformations of less than 2 μm.

Regarding the stiffness and strength of the instrument base and optical detection system, based on the simulation results, the instrument base’s maximum stress (0.99 MPa) occurred in the support beam, while the optical inspection subsystem’s maximum stress (0.59 MPa) was observed at the illumination connection and cover plate; both of these were well within the materials’ acceptable stress limits and fulfilled the design specifications.

#### 2.3.2. Modal Analysis

**Figure 8:**
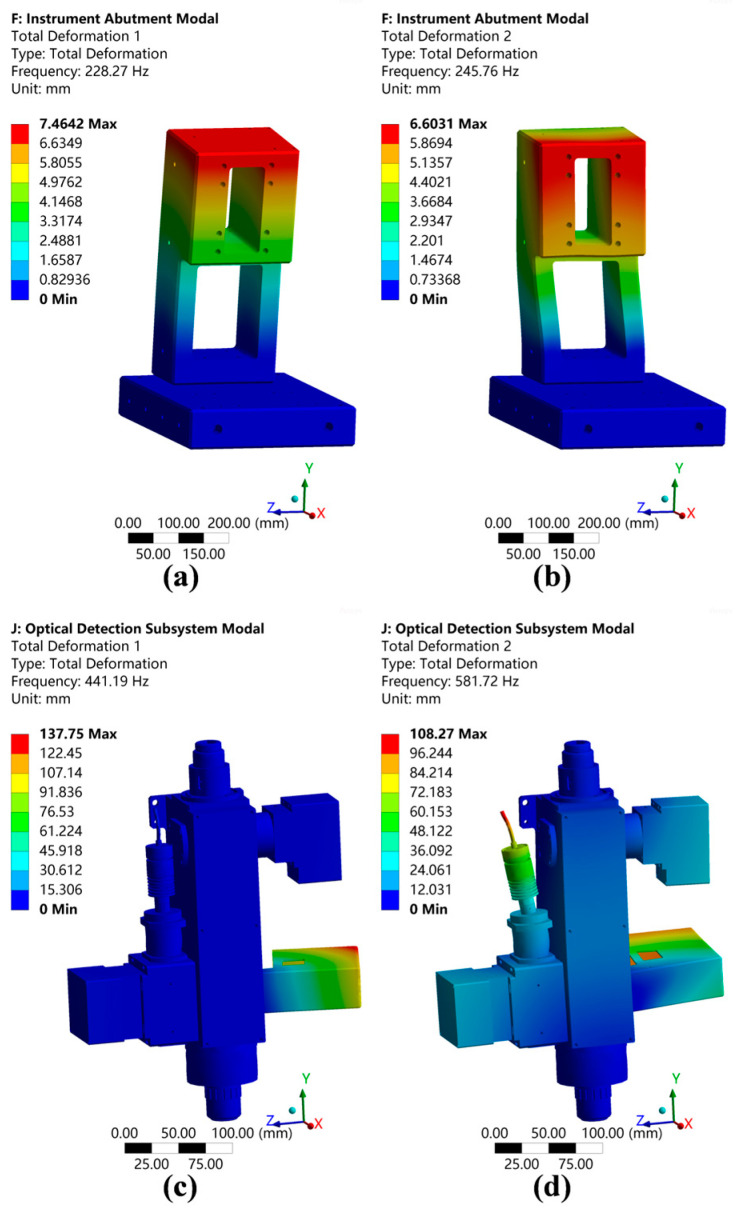
Modal simulation results. (**a**) Instrument base’s first-order vibration mode. (**b**) Instrument base’s second-order vibration mode. (**c**) Optical detection subsystem’s first-order vibration mode. (**d**) Optical detection subsystem’s second-order vibration mode.

To assess the structure’s dynamic stiffness further and mitigate potential resonance-related damage, a modal analysis of the structure was necessary, especially for the instrument base and optical inspection subsystem. The settings for the material, contact, constraints, and mesh were identical to those outlined in Section 2.3.1. During the analysis, damping effects were disregarded, which resulted in the acquisition of the first six mode shapes and natural frequencies without pre-stress. Figure 8 illustrates the structure’s mode shapes, while Table 3 provides the natural frequencies for each mode.

According to the simulation outcomes, the instrument’s base frequency was 228.27 Hz, while that of the optical inspection subsystem was 441.19 Hz. The entire system’s integrated fundamental frequency was no less than the lower of these two, which was 228.27 Hz, and thus, met the design requirements. The structure demonstrated excellent dynamic stiffness.

#### 2.3.3. Random Vibration Simulation

**Figure 9:**
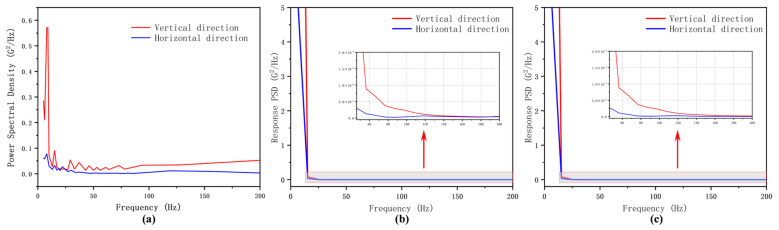
Power spectral density curves. (**a**) GJB150.16A specified the PSD curve. (**b**) The response PSD in the horizontal and vertical directions of the instrument base monitoring points. (**c**) The optical inspection subsystem monitored the response PSD in the horizontal and vertical directions of the monitoring points.

For the detection instrument, non-deterministic loads exist in non-laboratory environments, such as transportation. Therefore, to determine the structural dynamic stiffness, it was necessary to conduct random vibration simulations on the instrument. We referred to the test method for the laboratory environment of military equipment specified in the National Military Standard of the People’s Republic of China (GJB150.16A-2009) for trailer transportation power spectral density. The power spectral density curve is shown in Figure 9a. Analyzing the power spectral density spectrum with relatively strong excitation according to the military standard can reflect a more rigorous environment, thereby presenting a more comprehensive structural response range under random vibration excitation.

**Figure 10:**
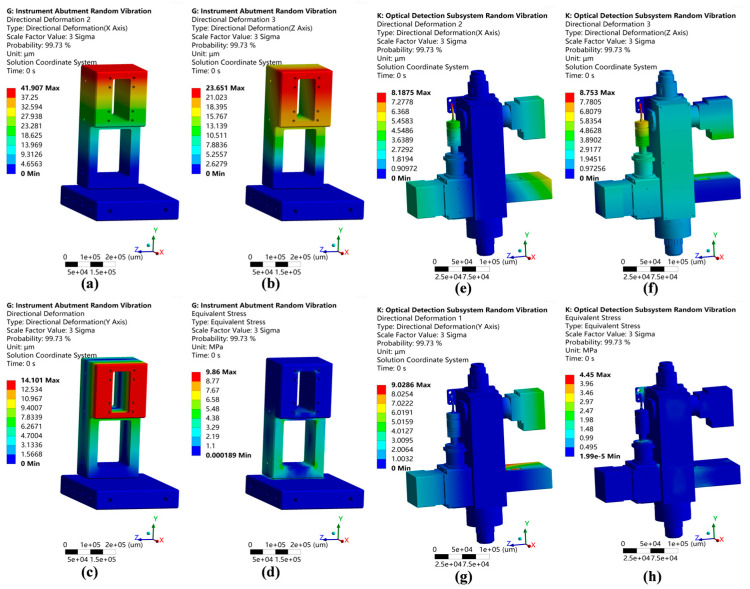
Random vibration simulation nephograms. (**a**–**c**) Instrument base’s deformation nephogram in the XYZ direction. (**d**) Instrument base’s equivalent stress nephogram. (**e**–**g**) Optical inspection subsystem XYZ-direction deformation nephogram. (**h**) Equivalent optical inspection subsystem’s stress nephogram.

According to Figure 9a, the input power spectral density was set in the vertical and horizontal directions for the structure by utilizing the modal superposition method to extract the natural frequencies and mode shape results obtained from the modal analysis in Section 2.3.2. Monitoring points were set at the instrument base mounting surface and the optical inspection subsystem detector. The response within the 3σ confidence interval was used as the result, where for a Gaussian distribution, 3σ corresponds to a probability of 99.73%. The simulation results are shown in Figure 10. The power spectral density curves of the monitoring points’ responses are illustrated in Figure 9.

In the simulated results, under harsh transportation conditions, the optical inspection subsystem experienced a maximum deformation of 9.02 μm in all directions, while the maximum deformation of the instrument base reached 41.907 μm. This may lead to changes in the performance of the instrument during transportation, thus requiring reinforcement and fixation of the base rear during transportation. The maximum stress on the instrument base (9.86 MPa) occurred at the support beam, while the maximum stress on the optical inspection subsystem (4.45 MPa) occurred at the mounting angle; both deformations fell within the materials’ acceptable stress limits, which satisfied the design specifications.

### 2.4. Prototype Development

**Figure 11:**
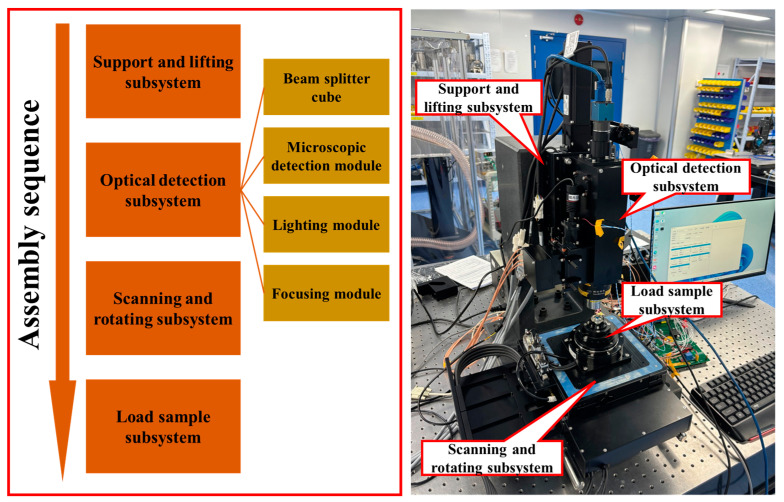
Steps and outcomes involved in assembling and adjusting the prototype.

The assembly and precision adjustment directly determine the overall accuracy and responsiveness of the machine during detection. The optical system was assembled and adjusted according to the process shown in Figure 11, with a focus on the optical inspection subsystem, including the focusing module, detector module, two beam splitters, and illumination module. The adjustment components were provided for each part’s precise adjustment, as described in Section 2.2.

The assembly and adjustment of the motorized displacement stage primarily involved adjusting the orthogonality between the horizontal displacement stage and the camera, as well as the relative position of the *Z*-axis lifting stage to the rotation stage center, to ensure accurate positioning during movement. The pitch adjustment stage was undertaken primarily to ensure the relative level of the sample surface to the focal plane to ensure that it did not exceed the focus depth during movement. In these steps, instruments such as the RENISHAW laser interferometer, TRIOPTICS angle measurement instrument, TRIOPTICS center deviation measurement instrument, dial gauge, and theodolite were required. The steps and outcomes involved in assembling and adjusting the prototype are shown in Figure 11.

## 3. Results

Patterned and unpatterned defects are two common types of defects in the semiconductor manufacturing and inspection fields. Patterned defects are related to the deposition, etching, printing, and other processing steps of the pattern, while non-patterned defects are unrelated to the pattern and may be caused by various factors.

### 3.1. Unpatterned Defect Detection Effectiveness

**Figure 12:**
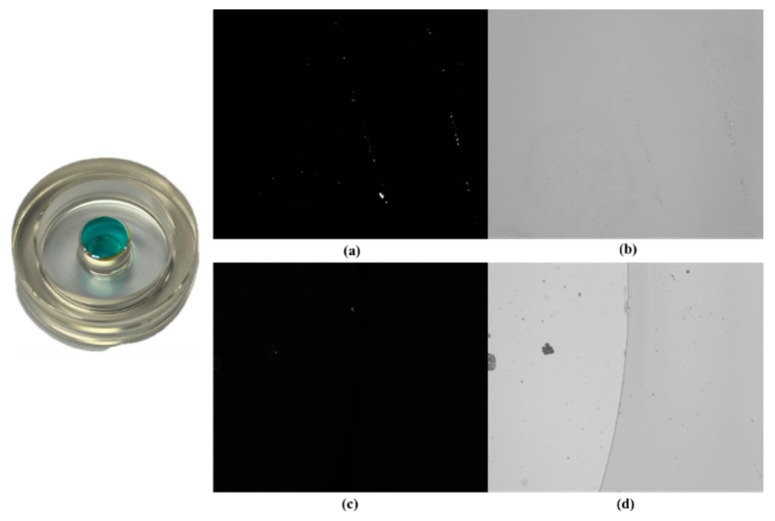
Laser gyro reflector substrate detection results. (**a**) Dark-field detection results in the coated area. (**b**) Bright-field detection results in the coated area. (**c**) Dark-field detection results in the optical adhesive (transparent) area. (**d**) Bright-field detection results in the optical adhesive (transparent) area.

To validate the prototype sensitivity in defect detection, the prototype shown in Figure 11 was used to inspect defective 25 mm laser gyroscope reflector substrates. This process confirmed the structural soundness of the prototype. The inspection results are shown in Figure 12, where Figure 12a shows imperfections, such as particles, scratches, contamination, and indentations, that were observed on the laser gyroscope surface.

The detection results indicate that in the sample’s optical adhesive (transparent) area, the dark-field laser illumination could cause some background noise interference from the substrate. Therefore, bright-field LED illumination was more suitable for sample inspection. For the sample’s coated area, both the dark-field and bright-field inspections yielded satisfactory results. The dark-field illumination was more sensitive to small defects and provided higher contrast, while the bright-field illumination offered clearer descriptions of the macroscopic defects. Therefore, they complemented each other. Due to the laboratory working environment being at the 10,000 level, unavoidable dust particles and other contaminants could have been distributed on the sample surface, in addition to standard defects.

### 3.2. Patterned Defect Detection Results

In the defect detection field, unpatterned and patterned defect detection often follow two distinct technical approaches. To validate the prototype’s performance and resolution for patterned defect detection, a quantitative assessment was conducted using the USAF 1951 resolution chart, which is a resolution test pattern that complies with the MIL-STD-150A standard created by the United States Air Force in 1951 and is widely used to test the resolution capability of optical imaging systems.

**Figure 13:**
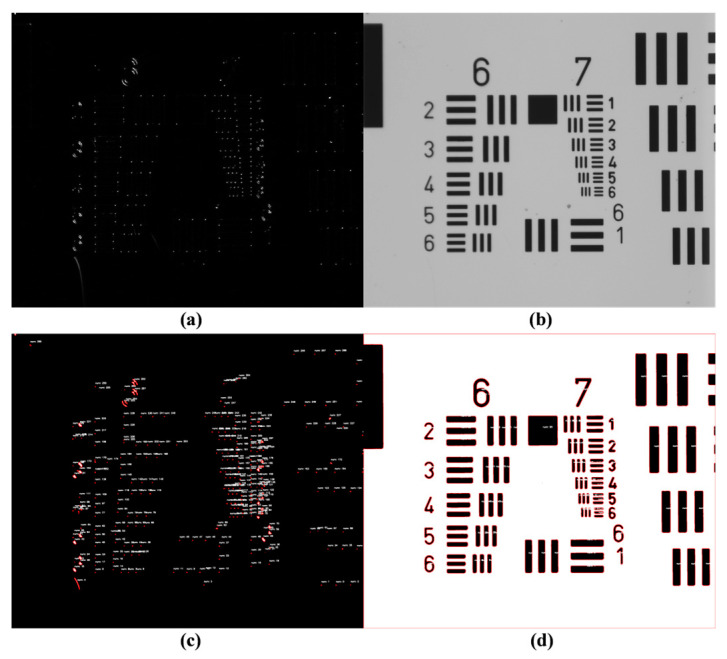
USAF 1951 test results. (**a**) Dark-field detection results. (**b**) Bright-field detection results. (**c**) Defect identification results in dark field. (**d**) Defect identification results in bright field.

The evaluation results are depicted in Figure 13, demonstrating the prototype’s defect detection resolution.

Figure 13a shows that the incident direction of the dark-field laser illumination correlated with the detection results for the patterned defect detection. Light incident parallel to the defect marks failed to produce scattered imaging of the defects (Figure 13c). This phenomenon significantly impacted the defect identification accuracy. To avoid missed and incorrect detections, at least two mutually perpendicular light source incident directions were required, which validated the necessity of the rotating stage and its calibration in our structural design.

**Figure 14:**
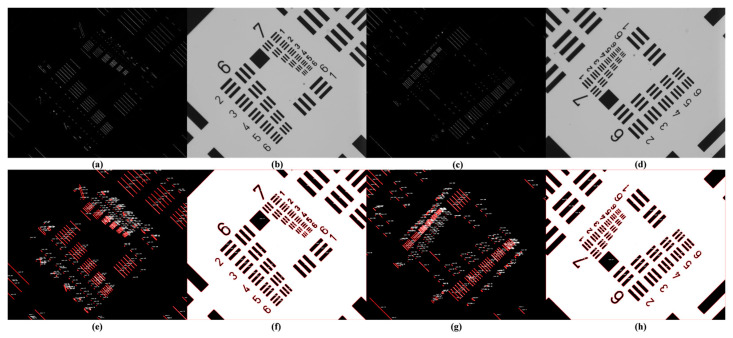
USAF 1951 rotation detection results. (**a**) Dark-field detection results after rotating 45°. (**b**) Bright-field detection results after rotating 45°. (**c**) Dark-field detection results after rotating 135°. (**d**) Bright-field detection results after rotating 135°. (**e**) Defect identification results of dark-field detection at 45° rotation. (**f**) Defect identification results of bright-field detection at 45° rotation. (**g**) Defect identification results of dark-field detection at 135° rotation. (**h**) Defect identification results of bright-field detection at 135° rotation.

We rotated the USAF 1951 sample by 45° and 135°, and the detection results are shown in Figure 14a–d. Under laser dark-field illumination, only the defects along the incident light’s normal direction scattered the light, while the radial defects did not. Therefore, for patterned defect detection, dark-field illumination alone was insufficient and bright-field processing was essential. Dark-field illumination can serve as an auxiliary for detecting small defects, providing a combined approach for a comprehensive analysis.

To evaluate the defect identification results after the binarization shown in Figure 14e–h, a comparison is made in Table 4, which details the number of line pairs per mm in the USAF Resolving Power Test Target 1951. The test chart consisted of a series of elements that appear as two sets of vertical lines. Each set was composed of three lines separated by spaces equal to the line width, which resulted in a 5:1 aspect ratio (where the line width was half the line spacing, which was the inverse of the line frequency). The elements were arranged in groups of six to form pairs. Even-numbered groups occupied the left and lower-right corners and included a square feature with a side length equal to the line length of the elements in that group. Odd-numbered groups occupied the upper-right and side edges. Groups and elements were identified and distinguished by the numbers adjacent to their features. The number of line pairs per mm in the USAF Resolving Power Test Target 1951 is shown in Table 4.

Here, we focused primarily on the data from groups 6 and 7. For defects on the Test Target with a true line length *L_true_* as follows:

(2) $$L_{true}=\frac{5}{2}\cdot LP,$$

where *LP* represents the line pairs in the USAF Resolving Power Test Target 1951:

(3) $$LP=\frac{1}{N}\;\mathrm{mm}=\frac{10^{6}}{N}\;\mathrm{nm},$$

where *N* represents the number of line pairs per mm in the USAF Resolving Power Test Target 1951. Taking the fourth and fifth elements of the sixth group as examples, the calculations yielded *L_true64_* = 27,624.3094 nm and *L_true65_* = 24,509.8039 nm. Table 5 shows the corresponding identified defect line average lengths $\bar{L}$*_dentify_*.

The single-pixel resolution ($\bar{RES}$) for each scenario was calculated to minimize the error by averaging using the following formula. The single-pixel resolution refers to the size of the smallest pixel that can be resolved without sub-pixel-level algorithm processing. *n* represents the number of pairs involved in the calculation.

(4) $$\bar{RES}=\frac{\sum_{k=1}^{n}RES}{n}=\frac{\sum_{k=1}^{n}\frac{L_{true}}{L_{identify}}}{n},$$

The calculation results were as follows:

$${\bar{RES}}_{45dark}\approx 180.230\;\mathrm{nm}$$

$${\bar{RES}}_{45bright}\approx 168.637\;\mathrm{nm}$$

$${\bar{RES}}_{135dark}\approx 184.752\;\mathrm{nm}$$

$${\bar{RES}}_{135bright}\approx 168.691\;\mathrm{nm}$$

Based on the results, the bright-field detection resolution was better than 170 nm. Additionally, for the patterned defect detection, the calculated resolution in the bright-field mode was superior to that in the dark-field mode. This was due to the diffusion effect of defects under the dark-field laser illumination, which caused the pixel size to appear larger after binarization, which resulted in a higher calculated resolution. This finding corroborated our earlier conclusion and explained why industrial applications primarily use bright-field detection for patterned defects, supplemented by dark-field detection.

## 4. Discussion

Compared with the instruments proposed by Jules Karangwa [23], Fan Wu [24], and Ding [22] for unpatterned defect detection, our instrument was capable of simultaneously detecting patterned and unpatterned defects. Additionally, in contrast to commercial defect detection instruments, such as the KLA-SP series operating under single working conditions, our instrument can perform integrated surface and edge defect detection. Furthermore, through structural innovation, our instrument can simultaneously detect reflective and transmissive surfaces, simplifying the production process and enhancing the operator efficiency compared with the aforementioned instruments.

However, based on the foundation of this defect detection prototype, there still exists room for improvement, including the following:

1. The KLA-SP series has proposed an integrated defect detection product with four channels. For this product, adding polarization and fluorescence detection channels may be considered, but this requires further optimization in the structure.
2. In addition to laser gyro detection, this defect detection prototype can also be applied in the semiconductor field. This includes unpatterned wafer detection and photomask template defect detection. However, modifications to the sample carrier design are required for different types of samples.
3. Considering the size characteristics of the investigated samples, the range of motion of the selected displacement stage was relatively small. The maximum applicable wafer size was only 4 inches, and the production rate was limited to around 4WPH. Therefore, if applied to semiconductor inspection, consideration should be given to replacing the displacement stage with a larger range of motion to detect larger wafers.
4. To meet various defect detection requirements, the structure features a quick-release design for the lens, allowing for an objective lens exchange with different magnification levels. Currently, the 170 nm detection resolution corresponds to a 20× magnification. At a 50× magnification, the detection resolution can be improved to 70 nm. However, increasing the detection resolution will inevitably reduce the detection efficiency. Therefore, different magnification levels can be reasonably selected based on specific detection needs, thereby accommodating various industrial requirements.

## 5. Conclusions

This paper introduces the core concept and structural implementation of a defect detection scheme, with a focus on the latter. In this study, we designed a dual-channel, multi-detector laser gyroscope mirror substrate defect detection prototype based on the same platform. Each module’s structural design was realized, and the components were designed to meet the assembly precision requirements and instrument functional needs, which culminated in the complete instrument. Additional verification of the structure’s feasibility was conducted through simulations and practical tests. The simulation outcomes confirmed that the structure aligned with the optical specifications and mechanical demands. During practical testing, the prototype successfully identified imperfections, such as particles, indentations, scratches, and contaminations, on the laser gyroscope mirror. Additionally, the detection resolution and patterned defect detection performance were verified using the USAF 1951 test target. These findings underscore the soundness of the prototype’s structural design and its suitability for detecting defects, providing a high-integration, cost-effective solution capable of simultaneous multi-type defect detection for the defect detection field.

## 6. Patents

We applied for a Chinese national invention patent for this design (patent number: CN202410163564.0).

## Figures and Tables

**Table 1:** Material definitions.

Material	Poisson’s Ratio	Young’s Modulus (GPa)	Density (kg·m^−3^)
Stainless steel	0.31	193	7750
Marble	0.26	121	2660
6061	0.33	69.04	2713
Fused quartz	0.17	70	2200
2A12	0.33	72.4	2770

**Table 2:** Deformation of key parts in optical detection subsystem.

	Horizontal Deformation (μm)			Vertical Deformation (Z-Direction) (μm)	Total Deformation (μm)
	X	Y	Total		
DVD read–write head (focusing module)	0.2157	0.34585	0.4076	0.31672	0.50829
CMOS (microscopic detection module)	0.0335	0.21635	0.2189	0.029368	0.21644
PMT (microscopic detection module)	0.2537	0.42412	0.4942	0.30718	0.57172
LED (bright-field lighting module)	0.0634	1.6041	1.6053	1.6117	0.20554
Laser turning mirror (dark-field lighting module)	0.2990	0.49841	0.5812	0.2996	0.62979

**Table 3:** Modal simulation results.

Modal	Instrument Base Natural Frequency (Hz)	Instrument Base Deformation Direction	Natural Frequency of Optical Inspection Subsystem (Hz)	The Position of Maximum Deformation
1	228.27	Overturned along the *X*-axis	441.19	Laser-turning mirror
2	245.76	Overturned along the *Y*-axis	581.72	LED and laser-turning mirror
3	504.19	Rotated around the *Z*-axis	614.95	Laser-turning mirror
4	1007.4	Overturned along the X- and Y-axes	711.78	LED
5	1687.3	Overturned along the *Y*-axis and rotated around the *Z*-axis	775.07	LED
6	2205.1	Overturned along the *X*-axis and rotated around the *Z*-axis	881.47	PMT

**Table 4:** Number of line pairs/mm in USAF Resolving Power Test Target 1951.

Element	Group Number										For High Res Only	
	−2	−1	0	1	2	3	4	5	6	7	8	9
1	0.250	0.500	1.00	2.00	4.00	8.00	16.00	32.0	64.0	128.0	256.0	512.0
2	0.280	0.561	1.12	2.24	4.49	8.98	17.95	36.0	71.8	144.0	287.0	575.0
3	0.315	0.630	1.26	2.52	5.04	10.10	20.16	40.3	80.6	161.0	323.0	645.0
4	0.353	0.707	1.41	2.83	5.66	11.30	22.62	45.3	90.5	181.0	362.0	—
5	0.397	0.793	1.59	3.17	6.35	12.70	25.39	50.8	102.0	203.0	406.0	—
6	0.445	0.891	1.78	3.56	7.13	14.30	28.50	57.0	114.0	228.0	456.0	—

**Table 5:** $\bar{L}$*_dentify_* at different rotation angles.

	$\bar{L}$*_dentify64_* (Pixels)	$\bar{L}$*_dentify65_* (Pixels)
Rotation 45° dark field	152.735	136.4716
Rotation 45° bright field	163.3417	145.7586
Rotation 135° dark field	149.2151	132.9361
Rotation 135° bright field	163.3417	145.664

## Data Availability

The datasets generated and/or analyzed during the current study are available from the corresponding author upon reasonable request.
